# Exploring the Application Value of Magnetocardiography in Detecting Pulmonary Hypertension: A Noninvasive and Visual Approach

**DOI:** 10.1002/clc.70277

**Published:** 2026-03-23

**Authors:** Yuankun Qi, Jiaqi Liang, Yu Zhang, Jianzhi Yang, Fuzhi Cao, Xu Zhang, Haijun Li, Xiaopei Cui, Hongyu Zhang, Min Xiang

**Affiliations:** ^1^ Key Laboratory of Ultra‐Weak Magnetic Field Measurement Technology, Ministry of Education, School of Instrumentation and Optoelectronic Engineering Beihang University Beijing China; ^2^ Hangzhou Innovation Institute Beihang University Hangzhou China; ^3^ Shandong Key Laboratory for Magnetic Field‐free Medicine & Functional Imaging, Institute of Magnetic Field‐free Medicine & Functional Imaging Shandong University Jinan Shandong China; ^4^ Department of Geriatric Medicine Qilu Hospital of Shandong University Jinan Shandong China; ^5^ Key Laboratory of Cardiovascular Proteomics of Shandong Province Jinan Shandong China; ^6^ Jinan Clinical Research Center for Geriatric Medicine Jinan Shandong China; ^7^ National Institute of Extremely‐weak Magnetic Field Infrastructure Hangzhou China; ^8^ Hefei National Laboratory Hefei China

**Keywords:** cardiac imaging techniques, magnetocardiography, pulmonary hypertension

## Abstract

**Background:**

Exploring accurate and noninvasive methods for detecting pulmonary hypertension (PH) has always been a focal point of research. Owing to its exceptional spatiotemporal resolution, magnetocardiography (MCG) has demonstrated potential value in cardiovascular diseases.

**Aims:**

This exploratory study aims to investigate the characteristics of MCG variations in PH patients and evaluate their potential utility in distinguishing healthy subjects from those with PH.

**Methods:**

This study analyzed 175 PH patients and 333 healthy subjects who underwent MCG examination. The training cohort consisted of patients with PH previously diagnosed by right heart catheterization (RHC) and age‐frequency‐matched healthy controls (HC). The testing cohort comprised age‐ and frequency‐matched HC and PH patients who underwent both MCG and RHC on the same day. Nine MCG parameters were included. Logistic regression was used to screen for significant parameters and develop a model.

**Results:**

By comparing the pseudo‐current density maps, it was found that the current vector at the R‐wave peak of HC points toward the lower‐left quadrant, whereas in PH patients, it points toward the lower‐right quadrant. The MCG detection model demonstrated robust performance, achieving a sensitivity of 86.1% and a specificity of 94.1% in the testing cohort. Compared to the ECG of PH patients, MCG demonstrated greater sensitivity; however, it exhibited slightly lower specificity. Furthermore, MCG can detect PH in patients with normal ECG findings.

**Conclusion:**

MCG demonstrates highly promising potential for the noninvasive detection of PH.

## Graphical abstract

MCG may serve as a noninvasive, visualized tool for detecting PH.

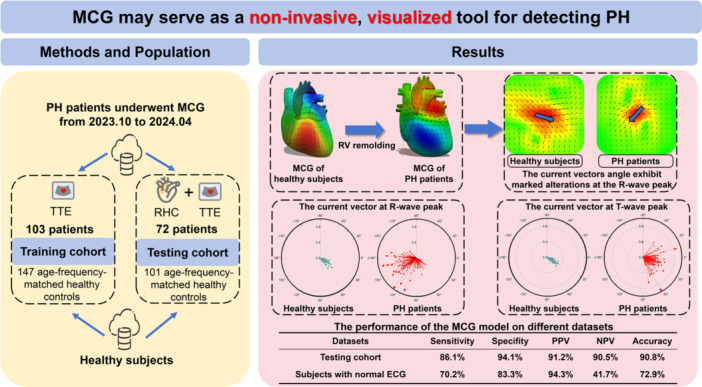

## 1 Introduction

Pulmonary hypertension (PH) is a progressive and life‐threatening disease affecting at least 1% of the global population [1]. Early diagnosis and intervention are critical for prolonging survival and enhancing quality of life in patients with PH. Right heart catheterization (RHC) remains the gold standard for diagnosis; yet it is an invasive method that may lead to various complications [2]. Moreover, RHC is a technically demanding procedure, often restricted to specialized pulmonary vascular centers due to its complexity. Over the past few decades, clinical researchers have persistently explored noninvasive detection methods for PH. Transthoracic echocardiography (TTE) is a noninvasive tool widely utilized in clinical practice. Although TTE can provide essential cardiac structural parameters and estimate pulmonary pressure, its accuracy is highly operator‐dependent. Furthermore, its reliability becomes questionable in cases of suboptimal or unobtainable tricuspid regurgitation signals [3, 4]. Therefore, there is an urgent need for an assessment method based on a novel principle that is intuitive, noninvasive, and operator‐independent.

Magnetocardiography (MCG) is a non‐contact technique that continuously records variations in magnetic fields (MF) (from 10^−11 ^T to 10^−14 ^T) throughout the cardiac cycle [5]. MCG provides higher spatial and temporal resolution than conventional ECG and is more sensitive to tangential and vortex currents which conventional ECG cannot detect [6, 7]. Furthermore, the propagation of cardiac MF within the human body remains unaffected by the lungs, adipose tissue, and skin [8]. Critically, the integrated software automatically generates quantitative parameters and graphical outputs, enabling rapid and standardized interpretation. Given the advantages of MCG, numerous studies have explored its role in coronary heart disease, arrhythmology, inflammatory cardiomyopathy, and fetal heart disease [9]. With the increase in pulmonary vascular resistance and mean pulmonary arterial pressure (mPAP), the right ventricle (RV) undergoes hypertrophy and dilation, leading to alterations in cardiac electrical activity, detectable with high resolution using MCG [6]. The aim of our study was to investigate the clinical value of MCG in the assessment of PH.

## 2 Methods

### 2.1 Study Population and Design

This was a single‐center, exploratory, proof‐of‐concept study. PH patients who underwent MCG between October 2023 and May 2024 were retrospectively reviewed, and categorized into two groups: (1) patients who underwent RHC (PH subgroup A); and (2) patients with a previous RHC confirmed diagnosis of PH who were followed up in the outpatient clinic using TTE and were assessed as having a high probability of PH (PH subgroup B). To ensure a more homogeneous patient cohort and minimize the confounding effects of left heart diseases, only patients with pre‐capillary PH (Group 1 and Group 4) were included. Patients with metallic implants were excluded due to the substantial signal noise generated by these devices, which compromises the accuracy of result interpretation. In addition, pregnant patients were excluded.

Healthy subjects who had undergone TTE prior to MCG to exclude PH and other cardiac pathologies served as controls. MCG data of healthy subjects were obtained from the dataset of the Institute of Magnetic Field‐free Medicine & Functional Imaging, Shandong University. Healthy subjects were categorized into two groups: young healthy controls (YC) aged 20–29 and healthy controls (HC) aged 30–60.

The training cohort comprised PH subgroup B patients along with age‐frequency‐matched HC, while the testing cohort comprised PH subgroup A patients with the age‐frequency‐matched HC.

This secondary analysis of the MCG data set complied with the principles of the Declaration of Helsinki and was approved by the Ethics Committee of Shandong University of Qilu Hospital (KYLL‐202404‐002‐1), and the requirement for informed consent was waived due to its retrospective nature.

### 2.2 RHC

RHC was performed using a 131F7 Swan‐Ganz catheter (Edwards, Life‐Sciences, Irvine, CA, USA) according to standard procedure [10]. According to the latest guidelines, the hemodynamic diagnostic criteria for pre‐capillary PH were defined as mPAP > 20 mmHg, pulmonary artery wedge pressure ≤ 15 mmHg, and pulmonary vascular resistance > 2 Wood units.

### 2.3 TTE and ECG

PH patients underwent TTE examination using the GE Vivid E95 echocardiography machine (GE, Vingmed Ultrasound, Horten, Norway). According to guidelines, parameters that reflect heart structure and right heart function were collected [11]. Furthermore, the values of tricuspid regurgitation velocity (TRV) and the presence of other TTE signs of PH were used to define the probability of PH. 12‐ Lead ECG recordings were obtained with the Beneheart R12 device (Shenzhen Mindray Bio‐Medical Electronics Co. Ltd.) and interpreted by two cardiologists.

Unlike PH patients who received comprehensive TTE and ECG reports, the TTE and ECG examination of HC were solely used to exclude cardiac diseases such as PH, heart failure, and valvular heart disease before the MCG examination. Therefore, these examination records were not entered into the medical record system, and the classification of healthy status was determined by professional physicians at the research center. As a result, detailed TTE and ECG parameters for HC were not available.

### 2.4 MCG

Details regarding the MCG equipment and the measurement workflow are provided in the Supporting Information S1: Figure S1 and Supporting Information Material Methods. The MCG examination was completed within 120 s, comprising a 90 s scanning period.

**Figure 1:**
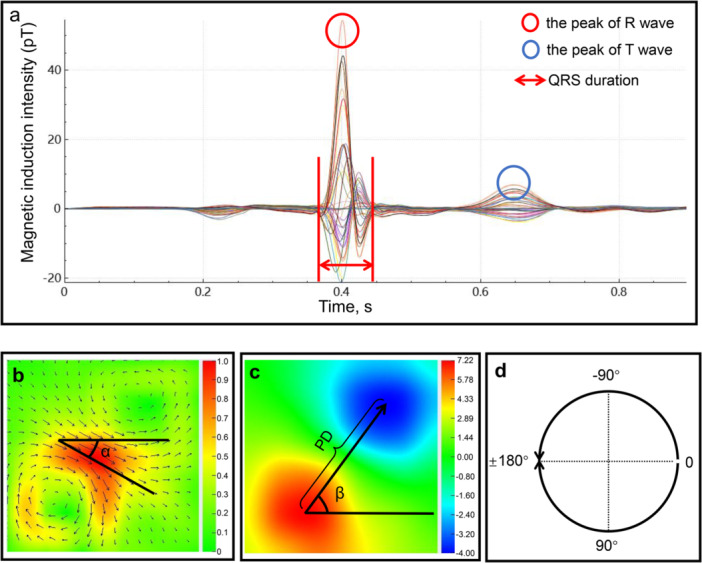
The “butterfly” diagram, pseudo‐current density (PCD) map, and magnetic field map (MFM) of a healthy male. (a) The “butterfly” diagram of a single cardiac cycle. (b) The PCD map at the T‐wave peak. *α* denotes the current angle. (c) The MF map at the T‐wave peak. *β* denotes the field map angle, and the poles distance indicates the distance between the red and blue poles. (d) A schematic diagram delineating the angle.

Nine MCG parameters were derived from the butterfly diagram (Figure 1a), the pseudo current density (PCD) map (Figure 1b), and the MF map (Figure 1c). The peak moment of the R‐wave or T‐wave was defined as the time point at which the single channel in the butterfly diagram reached its maximum amplitude during the R‐wave and T‐wave. The angular definition follows the conventions established in the ECG (Figure 1d).

1. QRSd: The duration of QRS complex. The unit of this parameter is expressed in milliseconds (ms).
2. NCD_R and NCD_T: The normalized current density at the R‐wave and T‐wave peak in the PCD maps. Due to the normalization process, these two parameters are dimensionless.
3. CA_R and CA_T: The current angle at the R‐wave and T‐wave peak in the PCD maps. The units of these two parameters are expressed in degrees.
4. PD_R and PD_T: The polar distance at the peak moments of the R‐wave and T‐wave in the MF maps. The units of these two parameters are expressed in pixels.
5. FMA_R and FMA_T: The field map angle at the R‐wave and T‐wave peak in the MF maps. The units of these two parameters are expressed in degrees.

### 2.5 Model Development

Due to limitations in sample size and the number of MCG parameters, complex machine learning methods may lead to overfitting and poor interpretability. Therefore, binary logistic regression was used for modeling in this study. Age and sex served as confounding factors and were forced into the model. Multicollinearity among variables was assessed before model fitting, and a variance inflation factor (VIF) less than 5 was acceptable. After adjustment for age and sex, MCG parameters were entered into the model using forward stepwise selection based on the likelihood ratio to derive the final model. The above process, including the determination of the optimal cut‐off value based on the Youden index, was conducted entirely in the training cohort. No model retraining or parameter adjustment was performed on the testing cohort.

The ECG model was developed using the Spiegelhalter‐Knill‐Jones method. The detailed procedures are provided in the Supporting Information Materials.

### 2.6 Statistical Analysis

Continuous variables were presented as the mean ± standard deviation (SD) if normally distributed, or the median and interquartile range (IQR) if not normally distributed. Categorical variables were expressed as counts and percentages (*n* [%]). For normally distributed data, when comparing the means of continuous variables between two independent groups, an independent samples *t*‐test was employed; for comparisons involving three or more independent groups, a one‐way analysis of variance (ANOVA) was utilized. Conversely, for non‐normally distributed data, the Mann−Whitney *U* test was used to compare the median of variables between two groups, while the Kruskal−Wallis *H* test was employed for comparisons involving more than two groups. If there was a significant difference between groups, a post hoc test was used to compare groups (Tukey HSD test for one‐way ANOVA and Bonferroni adjusted Dunn test for Kruskal−Wallis *H* test). Receiver operating characteristic (ROC) curves were used to assess the diagnostic performance of each parameter and determine the cut‐off values. To account for multiple comparisons in the univariable performance analyses, the Bonferroni correction was applied.

Performance metrics (sensitivity, specificity, positive predictive value [PPV], negative predictive value [NPV], and accuracy) were derived from the confusion matrix. The 95% confidence intervals (CI) were calculated using the Clopper−Pearson exact method. The paired diagnostic classifications obtained by MCG and ECG were compared using McNemar's test.

For all tests, *p* < 0.05 was considered statistically significant. Statistical analyses were performed using OriginPro (v. 2024 SR1, Origin Lab Corp., Northampton, MA, USA), Python (Version 3.12.0; Python Software Foundation, https://www.python.org), and SPSS (Version 27, IBM Corp., Chicago, IL, USA).

## 3 Results

### 3.1 Subjects and Cohort Characteristics

**Figure 2:**
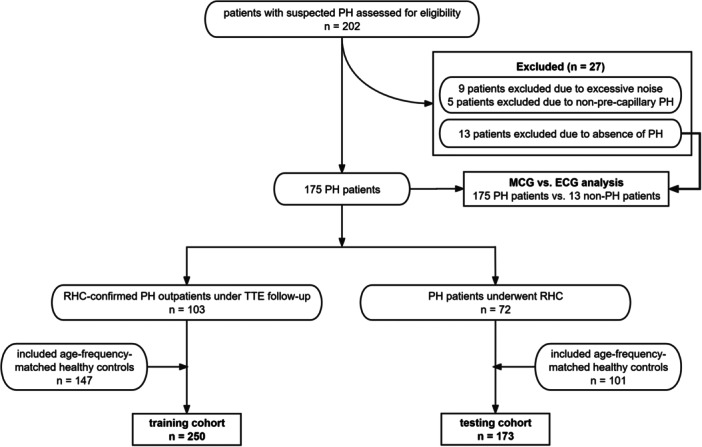
The flowchart of the study population and the analyzed cohorts.

A total of 202 patients with confirmed or suspected PH were initially identified. Fourteen patients were excluded for various reasons, and 13 were excluded after RHC examination (Figure 2). Consequently, 175 PH patients were ultimately included in the final analysis. Among these, 72 patients underwent RHC (PH subgroup A), while 103 underwent TTE follow‐up at the outpatient clinic (PH subgroup B). Additionally, 333 healthy subjects who had undergone MCG examination were included as controls, among whom 85 were classified as YC and 248 were classified as the HC.

The mean age of PH subgroup A patients was higher compared to PH subgroup B patients (46.92 ± 15.72 vs. 38.16 ± 12.01 years). This discrepancy is primarily attributed to a significantly higher proportion of chronic thromboembolic PH patients undergoing RHC compared with those followed in the outpatient clinic (30.6% vs. 5.8%) (Table 1a). This also resulted in a significant difference in subjects' age between the training and testing cohorts (Supporting Information S1: Table S1). All PH subgroup B patients received PH‐specific medications, and the median time since initial diagnosis was 34 months (Supporting Information S1: Table S2). Regarding the TTE and RHC parameters, RV outflow tract acceleration time and mean RV pressure exhibited significant differences between PH subgroup A and subgroup B patients (Table 1b).

**Table 1a:** Baseline demographic characteristics of PH patients.

Variables	All PH patients (*n* = 175)	Data for PH patients in		
		PH subgroup B (*n* = 103)	PH subgroup A (*n* = 72)	*p* value
Age, years	41.76 ± 14.29	38.16 ± 12.01	46.92 ± 15.72	< 0.001
Female, *N* (%)	141 (80.6%)	83 (80.6%)	58 (80.6%)	0.996
BMI, kg/m^2^	22.72 ± 3.51	22.16 ± 3.43	23.52 ± 3.50	0.011
6MWD, m	464.31 ± 83.77	474.83 ± 91.97	449.26 ± 68.24	0.047
WHO FC				< 0.001
Ⅰ	25 (14.3%)	22 (21.4%)	3 (4.2%)	
Ⅱ	94 (53.8%)	64 (62.2%)	30 (41.7%)	
Ⅲ	54 (30.9%)	17 (16.5%)	37 (51.4%)	
Ⅳ	2 (1.1%)	0	2 (2.8%)	
NT‐proBNP, pg/mL	430.00 (117.73, 763.45)	465.35 (116.90, 541.70)	406.00 (119.75, 1547.50)	0.110
Etiology, *N* (%)				< 0.001
I/HPAH	52 (29.7%)	35 (34.0%)	17 (23.6%)	
CHD‐PAH	57 (33.7%)	37 (36.9%)	20 (27.8%)	
Shunt correction	30 (52.6%)	25 (67.6%)	5 (25.0%)	
CTD‐PAH	34 (18.9%)	24 (22.3%)	10 (13.9%)	
PoPH	2 (1.1%)	1 (1.0%)	1 (1.4%)	
PVOD/PCH‐PAH	2 (1.1%)	0	2 (2.8%)	
CTEPH	28 (15.4%)	6 (5.8%)	22 (30.6%)	

Abbreviations: BMI, body weight index; CHD‐PAH, congenital heart disease‐associated pulmonary arterial hypertension; CTD‐PAH, connective tissue disease‐associated pulmonary arterial hypertension; CTEPH, chronic thromboembolic pulmonary hypertension; I/HPAH, idiopathic or heritable pulmonary arterial hypertension; NT‐proBNP, N‐terminal pro‐brain natriuretic peptide; PoPH, porto‐pulmonary hypertension; PVOD/PCH‐PAH, pulmonary arterial hypertension associated with pulmonary veno‐occlusive disease or capillary haemangiomatosis; WHO‐FC, World Health Organization functional class; 6MWD, 6‐minute walk distance.

**Table 1b:** The TTE and RHC parameters of PH patients.

Parameters	All PH patients (*n* = 175)	Data for PH patients in		
		PH subgroup B (*n* = 103)	PH subgroup A (*n* = 72)	*p* value
TTE parameters				
LVD, mm	37.16 ± 16.90	35.76 ± 8.34	36.39 ± 9.05	0.654
RAA, mm^2^	18.00 (14.00, 23.00)	17.00 (13.00, 23.00)	18.00 (14.00, 23.75)	0.222
RVD, mm	31.91 ± 9.35	31.29 ± 9.08	32.78 ± 9.71	0.304
RV/LV ratio	0.79 (0.59, 1.30)	0.78 (0.60, 1.18)	0.80 (0.57, 1.32)	0.951
mPA, mm	32.33 ± 9.00	33.08 ± 9.01	31.26 ± 8.93	0.191
IVC_E_, mm	15.86 ± 3.99	15.86 ± 3.96	15.86 ± 4.06	0.995
LVEI ratio	1.42 (0.98, 1.88)	1.51 (0.99, 1.96)	1.38 (0.97, 1.84)	0.188
TAPSE, mm	18.23 ± 3.52	18.23 ± 3.47	18.24 ± 3.61	0.973
RVOT‐AT, ms	69.26 ± 12.43	70.93 ± 12.96	66.88 ± 11.31	0.034
S', cm/s	12.14 ± 2.88	12.27 ± 2.87	11.94 ± 2.90	0.461
RIMP ratio	0.52 ± 0.19	0.51 ± 0.19	0.52 ± 0.18	0.667
TRV, m/s	4.20 ± 0.78	4.21 ± 0.82	4.19 ± 0.71	0.836
sPAP, mmHg	77.31 ± 28.09	77.81 ± 30.25	73.49 ± 26.81	0.779
TAPSE/sPAP, mm/mmHg	0.25 (0.18, 0.34)	0.25 (0.18, 0.37)	0.24 (0.19, 0.31)	0.659
RHC parameters				
mRAP, mmHg	4.00 (2.00, 5.00)	4.00 (2.75, 5.25)	3.00 (2.00, 5.00)	0.065
mRVP, mmHg	26.28 ± 10.42	28.55 ± 11.21	23.59 ± 8.74	0.007
mPAP, mmHg	47.03 ± 17.53	49.23 ± 17.95	44.76 ± 16.91	0.124
PAWP, mmHg	6.00 (4.00, 7.50)	6.00 (4.50, 8.00)	5.00 (4.00, 7.00)	0.062
CO, L/min	4.83 ± 1.59	4.70 ± 1.45	4.96 ± 1.73	0.332
CI, L/(min*Kg/m^2^)	2.99 ± 0.90	2.96 ± 0.82	3.01 ± 0.99	0.723
PVR, Wood units	7.71 (4.74, 12.04)	8.29 (5.21, 12.61)	7.11 (3.82, 11.07)	0.078
SvO_2_, %	67.13 ± 10.31	67.88 ± 9.13	66.38 ± 11.40	0.388

Abbreviations: CI, cardiac index; CO, cardiac output; IVC_E_, inferior vena cava diameter of end‐expiratory; LVD, left ventricular diameters; LVEI, left ventricular eccentricity index; mPA, main pulmonary arterial diameters; mPAP, mean pulmonary arterial pressure; mRAP, mean right atrial pressure; mRVP, mean right ventricular pressure; PAWP, pulmonary arterial wedge pressure; PVR, pulmonary vascular resistance; RAA, right atrial area; RIMP, right ventricular index of myocardial performance; RVD, right ventricular diameters; RVOT‐AT, right ventricular outflow tract‐acceleration time; S', tricuspid annular peak systolic velocity; sPAP, systolic pulmonary artery pressure; SvO_2_, mixed venous oxygen saturation; TAPSE, tricuspid annular plane systolic excursion; TRV, tricuspid regurgitation velocity.

The MCG parameters of PH patients exhibited significant differences compared to those of YC and HC (all *p* < 0.001), with the exception of QRSd. NCD_T, PD_R, PD_T, and FMA_R demonstrated significant differences between YC and HC (all *p* < 0.05). With advancing age, the current density at the T‐wave peak diminishes, whereas PD_R and PD_T demonstrated a progressive upward trend (Supporting Information S1: Table S3).

### 3.2 Alteration of Pseudo‐Current Density Maps in Patients With PH

**Figure 3:**
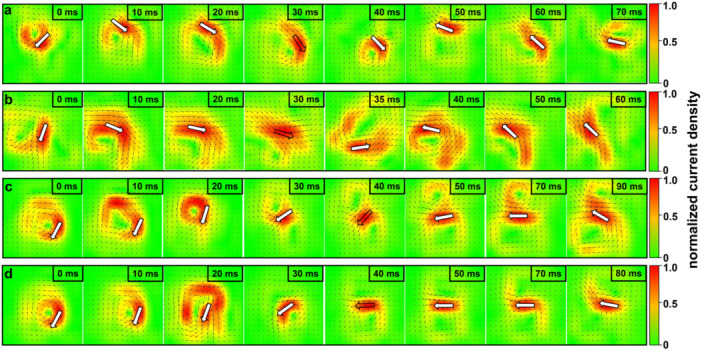
Current angle in PCD maps of HC and PH patients over time in the QRS interval. The small black arrows indicate the local current direction at each spatial location. The large white arrows (vector‐summed) represent the total current vector angle, whereas black frames represent the angle at the R‐wave peak. The color of maps represents the normalized current density (low, green; high, red). The PCD maps of a healthy female and male are presented in (a) and (b), respectively; whereas (c) and (d) show a female and male with PH, respectively. Abnormal deflection pattern (angle > 62°, 97.5th percentile of HC): PH 79.4% (139/175), HC 3.2% (8/248).

Compared with HC, the PCD maps of PH patients revealed an altered current pattern at the R‐wave peak, especially in terms of orientation. Figure 3a,b depict the PCD maps of a healthy female and a healthy male, respectively, with CA_R directed toward the lower‐left quadrant. In contrast, the CA_R in PH patients was oriented toward the lower‐right quadrant, as shown in Figure 3c,d. Meanwhile, the direction of the current was consistent between PH patients and HC at both the onset and offset of the QRS complex.

**Figure 4:**
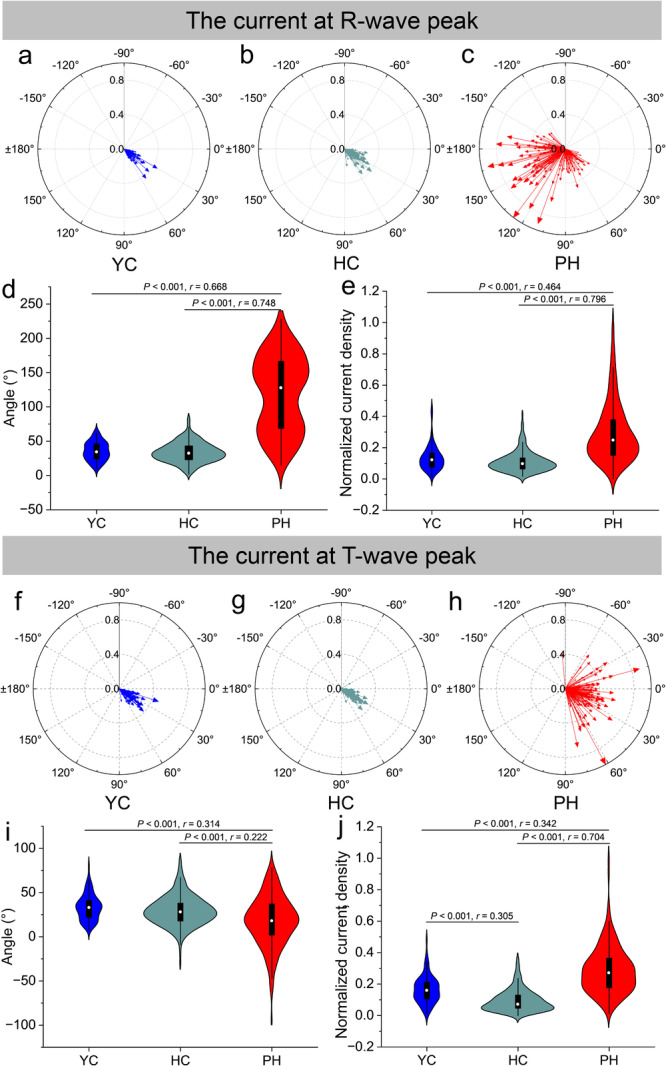
Vector diagrams for the R‐wave peak are shown in (a–c), and (d, e) present violin plots of the current angle and normalized current density at the R‐wave peak for YC, HC, and PH patients. (f–h) Show vector diagrams at the T‐wave peak, and panels (i, j) present violin plots of the current angle and normalized current density at the T‐wave peak for the same groups. The *r* in (d, e, i, j) means effect size *r*.

To provide a more intuitive visualization of the intergroup differences in the current vector among YC, HC, and PH patients, vector diagrams and violin plots were constructed. The vector diagrams demonstrated that compared with HC and YC, PH patients exhibited a distinct deflection in CA_R, which was predominantly localized in the third quadrant. Moreover, the NCD_R of PH patients was larger than that of HC and YC (Figure 4a–c). Intergroup comparisons showed that the CA_R of PH patients differed significantly from HC and YC, with large effect sizes (both *r* exceeding 0.5). For NCD_R, only PH patients and HC showed large effect sizes (Figure 4d,e). At the T‐wave peak, the current angle of PH patients exhibited greater dispersion compared to the other two groups, and significant statistical differences were also observed in NCD_T (Figure 4f–j). Furthermore, the NCD_T of the HC and YC groups demonstrated a significant difference, suggesting that the current density may be influenced by age.

### 3.3 The Potential of MCG for the Detection of PH

To evaluate the performance of single MCG parameters for detecting PH, the testing cohort was used to confirm the detection accuracy with the cut‐off values derived from the training cohort via ROC analysis. Results indicated that the sensitivity of the MCG parameters was suboptimal, with the exception of PD_T, which reached 79.5% (Supporting Information S1: Table S4). Conversely, specificity was notably high, with CA_R reaching 96.8%, and FMA_R and NCD_T both exceeding 90.0%. In terms of PPV and NPV, CA_R, NCD_T, FMA_R, and PD_T each exceeded 80%.

Prior to logistic regression modeling, collinearity diagnostics showed substantial multicollinearity between CA_R (VIF = 13.551, tolerance = 0.074) and FMA_R (VIF = 13.735, tolerance = 0.073). No significant multicollinearity was observed among the remaining parameters (Supporting Information S1: Table S5). CA_R is more intuitive, clinical interpretation, and its model yielded a lower −2 Log‐Likelihood than FMA_R. Moreover, there was no significant difference in AUC between the models in the training cohort (Supporting Information S1: Table S6). Therefore, CA_R was ultimately selected as the final parameter for modeling.

After adjusting for age and sex, NCD_T, CA_R, and PD_R exhibited predictive capabilities. The regression coefficients and odds ratios for this model are presented in Table 2. These five parameters were then utilized to construct a detection model. The Hosmer−Lemeshow test showed no significant difference between the observed and predicted probabilities in the training cohort (*χ* ^2^ = 4.070, df = 8, and *p *= 0.851). The VIF values for all parameters in the final model are presented in Supporting Information S1: Table S5, and the complete model with all original parameters is presented in Supporting Information S1: Table S7.

**Table 2:** The result of multivariable logistic regression.

Parameters	B	95% Bootstrap CI for B	OR (95% CI)	*p* value
Sex	−1.372	−3.246 to −0.357	0.254 (0.067 to 0.962)	0.044
Age	−0.010	−0.097 to 0.075	0.990 (0.932 to 1.053)	0.758
CA_R^a^	0.801	0.581 to 1.532	2.229 (1.574 to 3.156)	< 0.001
PD_R	−0.076	−0.169 to −0.035	0.927 (0.886 to 0.969)	< 0.001
NCD_T	0.136	0.062 to 0.277	1.146 (1.065 to 1.233)	< 0.001
Constant	3.266	−4.150 to 13.729	26.208	0.340

Abbreviations: B, regression coefficient; CI, confidence interval; OR, odds ratios.

^a^ In order to ease understanding and clinical application, the CA_R was scaled in increments of 10° during the modeling.

**Figure 5:**
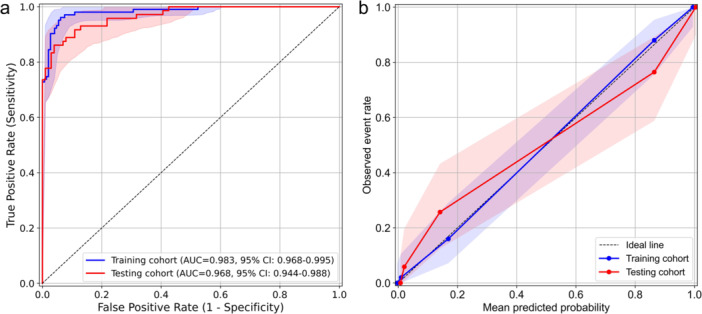
The ROC and calibration curves of MCG model in the training cohort and testing cohort. (a) ROC curves of the MCG model in the training and testing cohorts. The shaded areas present the 95% CI estimated by bootstrap resampling. (b) Calibration curves of MCG model in the training and testing cohort (the number of bins is 5, quantile). The shaded areas present the 95% CI.

In the training cohort, the apparent AUC of the logistic regression model was 0.983 (95% CI: 0.968–0.995). After bootstrap internal validation with 2000 resamples, the optimism‐corrected AUC was 0.977 (95% CI: 0.963–0.991). The AUC of this model in the testing cohort reached 0.968 (95% CI: 0.944–0.988) (Figure 5a). The optimal cut‐off value of the model was determined in the training cohort using the Youden index. Applying this cut‐off to the testing cohort resulted in lower sensitivity than in the training cohort (86.1% vs. 92.9%), whereas the specificity showed a slight increase (94.1% vs. 91.5%) (Table 3). The calibration curve showed good agreement between predicted and observed event probabilities in the training cohort. In contrast, the calibration curve in the testing cohort deviated more noticeably from the ideal line and had a wider confidence band, suggesting greater uncertainty in the model's predictions (Figure 5b).

**Table 3:** Performance of the MCG model in training cohort and testing cohort.

Cohorts	Sen (95% CI)	Spe (95% CI)	PPV (95% CI)	NPV (95% CI)	Acc (95% CI)
Training cohort (apparent)	96.1% (90.4%–98.9%)	93.9% (88.7%–97.2%)	91.7% (84.8%–96.1%)	97.2% (92.9%–99.2%)	94.8% (91.3%–97.2%)
Training cohort (optimism‐corrected)	92.9% (87.4%–97.4%)	91.5% (87.7%–94.5%)	88.5% (83.3%–93.0%)	95.0% (91.3%–98.3%)	92.1% (89.2%–94.8%)
Testing cohort	86.1% (75.9%–93.1%)	94.1% (87.5%–97.8%)	91.2% (81.8%–96.7%)	90.5% (83.2%–95.3%)	90.8% (85.4%–94.6%)

Abbreviations: Acc, accuracy; NPV, negative predictive value; PPV, positive predictive value; Sen, sensitivity; Spe, specificity.

### 3.4 The Sensitivity Analyses of the MCG Model

To evaluate the performance of the MCG model in different clinical scenarios, we performed various sensitivity analyses using the same model cut‐off value.

1. PAH versus CTEPH. The sensitivities of the model in the two PH Groups were 95.9% (142/148, 91.4%–98.5%) and 70.4% (19/27, 49.8%–86.2%) (*p* < 0.001).
2. To determine whether obesity (defined as BMI ≥ 28 kg/m^2^) influences the performance of the MCG model, a stratified sensitivity analysis was conducted. The sensitivity and specificity of the model in subjects without obesity (*n* = 411) were 91.5% (86.2%–95.3%) and 93.9% (90.1%–96.5%); and in subjects with obesity (*n* = 12) were 90.0% (55.5%–99.7%) and 100% (15.8%–100%) (all *p* > 0.05).
3. In the testing cohort, there were 33 patients were treatment‐naive. To evaluate whether treatment status (treated vs. treatment naive) had a potential impact on the model's performance, we conducted a subgroup analysis. The results demonstrated that the sensitivity of untreated PH patients was 90.9% (30/33, 75.7%–98.1%), whereas that in treated patients was 92.3% (131/142, 86.6%–96.1%) (*p* = 0.730).

### 3.5 Correlations Between MCG and Other Examinations

Compared with TTE parameters, CA_R, FMA_R, and NCD_R exhibited moderate correlations with the tricuspid annular plane systolic excursion to systolic pulmonary arterial pressure ratio and TRV, while FMA_R and NCD_R correlated moderately with the left ventricular eccentricity index. No significant correlation was found between MCG and the 6‐min walking distance (Supporting Information S1: Figure S2a). The relationship between MCG and RHC parameters revealed that CA_R and FMA_R were moderately correlated with pulmonary vascular resistance and mPAP. NCD_R exhibited moderate correlations with both mPAP and mean RV pressure, with the strongest correlation observed between NCD_R and mPAP (*r *= 0.6) (Supporting Information S1: Figure S2b).

### 3.6 Comparison of MCG and ECG in PH Detection

A comparative analysis of MCG and ECG was performed among 175 PH patients and 13 controls in whom PH was excluded (Figure 2). In the 175 PH patients, right‐axis deviation was the most predominant ECG abnormality (*n* = 111, 63.4%, Supporting Information S1: Table S8). Results indicated that the MCG model achieved significantly higher sensitivity for PH detection compared to the ECG model (91.4% vs. 73.1%, *p* < 0.001), albeit with a marginally reduced specificity (84.6% vs. 92.3%, *p *= 1.000). The paired comparison of diagnostic performance between MCG and ECG was assessed via McNemar's exact test (Supporting Information S1: Table S9). Among the 59 subjects with normal ECG findings, the MCG model retained a reliable diagnostic capacity, yielding a sensitivity of 70.2% and a specificity of 83.3% (Table 4).

**Table 4:** The capability comparison of MCG and ECG in PH detection.

Model	Sen (95% CI)	Spe (95% CI)	PPV (95% CI)	NPV (95% CI)	Acc (95% CI)
All subjects (*n* = 188)					
MCG model	91.4% (86.3%–95.1%)	84.6% (54.6%–98.1%)	98.8% (95.6%–99.9%)	42.3% (23.4%–63.1%)	91.0% (85.9%–94.6%)
ECG model	73.1% (65.9%–79.6%)	92.3% (64.0%–99.8%)	99.2% (95.8%–100.0%)	20.3% (11.0%–32.8%)	74.5% (67.6%–80.5%)
With normal ECG (*n* = 59)					
MCG model	70.2% (55.1%–82.7%)	83.3% (51.6%–97.9%)	94.3% (80.8%–99.3%)	41.7% (22.1%–63.4%)	72.9% (59.7%–83.6%)

Abbreviations: Acc, accuracy; CI, confidence interval; NPV, negative predictive value; PPV, positive predictive value; RAD, right‐axis deviation; RVH, right ventricular hypertrophy; Sen, sensitivity; Spe, specificity.

## 4 Discussion

Since its introduction in the 1970s, MCG has consistently proven its substantial clinical utility in detecting myocardial ischemia, fetal cardiac anomalies, arrhythmias, and assessing therapeutic efficacy in inflammatory cardiomyopathies [12, 13, 14, 15, 16, 17]. This study demonstrated that contactless MCG is an effective, noninvasive modality for detecting cardiac electrophysiological abnormalities, thereby enabling the identification of PH patients.

### 4.1 Clinical Implementation Considerations

The MCG system used in this study was based on Spin‐Exchange Relaxation‐Free optically pumped magnetometer (SERF‐OPM) technology. Compared with traditional superconducting quantum interference device (SQUID) MCG, it eliminates the need for liquid helium cooling and heavy magnetically shielded rooms, thereby significantly reducing both construction and operational costs. The entire workflow is accomplished within 3–4 min, with automated report generation that bypasses the need for a steep learning curve. Compared to TTE, MCG can provide a comprehensive visualization of both electrical and magnetic cardiac activities while significantly reducing operator dependency. Given these advantages, SERF‐OPM MCG technology is being actively investigated by numerous clinical centers worldwide [18, 19], representing a promising frontier in noninvasive cardiovascular detection. However, due to the inherent principles of SERF‐OPM, any metallic objects introduced into the magnetically shielded device can generate substantial noise artifacts. Consequently, this limitation precludes a group of patients from undergoing this examination.

We acknowledge that TTE remains the first‐line noninvasive modality for PH assessment. The principal utility of MCG resides in patients presenting with clinical manifestations of right heart dysfunction, including exertional dyspnea, fatigue, and hemoptysis. This becomes especially valuable in scenarios where TTE fails to provide an accurate assessment of PH, such as in cases with absent tricuspid regurgitation or when clinician expertise is limited.

### 4.2 Pathophysiological Basis for MCG in PH Detection

Our findings revealed a pronounced shift in the current vector among PH patients as observed via MCG. In healthy subjects, the current vector during the R‐wave peak predominantly aligns toward the lower left quadrant, corroborating findings from prior studies [20]. Conversely, in PH patients, the current vector tends to deviate toward the lower right quadrant. This alteration primarily stems from a transition in the overall electrocardial vector from left ventricular dominance to RV dominance during peak myocardial depolarization in PH patients, a phenomenon more visually apparent in MCG compared to ECG. With the progression of PH, elevated pulmonary vascular resistance and mPAP impose an increased afterload on the RV, culminating in RV hypertrophy (RVH) and dilation. Extensive research has established that ventricular hypertrophy and dilation augment ion exchange within cardiomyocytes, thereby amplifying transcellular currents [21, 22, 23]. In accordance with the Biot‐Savart and Ampère's Law, augmented transcellular currents directly enhance myocardial magnetic induction intensity. Furthermore, myocardial magnetic induction intensity depends strongly on the source‐to‐sensor distance, facilitating the precise detection of subtle alterations via MCG. The distinctive properties of MCG allow for the detection of nuanced electrical variations in cardiac activity, potentially indicating early‐stage heart diseases and surpassing the diagnostic precision of ECG.

Although the current vector shift may not be specific to PH and may instead reflect chronic RV pathology more broadly, it remains clinically useful. Among the patients with RV disorders, PH is the most common cause of chronic RV pathology, making these parameters potentially useful markers of RV remodeling. Future studies should systematically compare the MCG parameters between PH and other RV disorders, such as RV infarction, arrhythmogenic RV cardiomyopathy, and chronic left heart failure with secondary RV dysfunction. In addition, the findings of MCG should be interpreted within a clinical context rather than in isolation. MCG may serve as a useful complementary tool for noninvasive detection, but it should be considered together with clinical presentation, TTE findings, and other relevant examinations.

### 4.3 OPM‐MCG Diagnostic Performance in PH

In this study, a logistic regression model was developed by incorporating CA_R, NCD_T, and PD_R, after adjusting for age and sex. Despite optimism correction via 2000 bootstrap resamples, the model remained highly discriminative in the training cohort, with an AUC of 0.977. Furthermore, it demonstrated robust performance in the testing cohort, maintaining an AUC of 0.968. However, we acknowledge that the use of healthy subjects as the control group may overestimate the model's performance in both the training and testing cohorts.

For the three parameters in the final logistic regression model, the implications of CA_R have been discussed above. We hypothesize that variations in PD_R are associated with the distance between the sensor array and the equivalent current source of the heart. A shorter polar distance suggests that the current source is situated close to the sensors. In PH patients with RVH, the RV‐dominant equivalent current source may be positioned closer to the sensor panel (RV lies immediately behind the sternum), potentially leading to a reduction in PD_R. The PH‐induced increase in RV afterload impacts not only myocardial depolarization but also repolarization. Results revealed that NCD_T from the diastolic phase also holds predictive significance.

Our research indicates that MCG exhibited stability in sensitivity and accuracy compared to ECG. Especially when the ECG presents normal results, MCG retained its capacity for the detection of PH. This may be attributed to the high spatiotemporal sensitivity of MCG, as well as the minimal attenuation of MF signals during conduction in the human body. Given that the electrical signals associated with early lesions are relatively weak, they may undergo attenuation through human tissues and further filtering by the machine, leading to ineffective detection.

The MCG model demonstrated stable performance regardless of disease duration or treatment status. Although the training cohort included more patients with longer disease duration than the testing cohort, the model's discriminative ability remained consistent. In addition, the inclusion of patients receiving PH‐specific medications did not materially affect the results compared with treatment‐naive patients. The relatively small sample size may limit the statistical power. These findings further underscore the potential of MCG as a tool for the detection of PH.

The results of the correlation analysis showed that the NCD_R had the strongest correlation with mPAP among all parameters. Therefore, we hypothesize that NCD_R may be associated with ventricular loading. Given the moderate correlation observed, NCD_R might partially reflect the downstream consequences of ventricular hypertrophy secondary to increased loading conditions. Moreover, we propose that NCD_R in combination with CA_R may enable effective differentiation between the left and right ventricles [24, 25, 26]. This requires further validation by expanding the population of subjects.

## 5 Limitations

This study has several limitations that should be acknowledged, particularly as an exploratory, proof‐of‐concept study.

First, this was a single‐center retrospective study, with a limited sample size of patients undergoing RHC and MCG simultaneously. As a result, the training cohort comprised patients with PH previously diagnosed via RHC who were subsequently followed up with TTE, a potential source of bias that may have impacted the diagnostic model's performance. Despite having performed many sensitivity analyses, the small sample size may compromise the statistical power. Further validation through large‐scale multicenter research is essential prior to the routine clinical application.

Second, the reliability of MCG in PH detection remains to be fully established. As a novel technology, consistency analysis is critical prior to widespread clinical application. This includes inter‐device consistency and test‐retest reliability. These assessments are planned as integral components of the next prospective research.

Third, ECG and TTE examinations of healthy subjects were unavailable. This resulted in an insufficient number of negative samples during the performance comparison of ECG and MCG. Consequently, the prevalence‐dependent PPV and NPV may have been less representative. Moreover, the inclusion of healthy subjects in the control group may have created an artificially optimized scenario, potentially inflating the observed discriminative capacity of MCG for PH detection. In order to define the real‐world performance of MCG, future prospective studies should prioritize the recruitment of newly diagnosed PH patients, especially those with mild PH, and symptomatic controls with similar presentations to PH.

Finally, this study included a heterogeneous cohort of pre‐capillary PH (Group 1 and Group 4). Although sensitivity analysis showed differences in the model's sensitivity between the two groups, these results warrant further validation due to the limited sample size of CTEPH patients. Furthermore, future research will include PH associated with left heart disease (Group 2), lung disease/hypoxia (Group 3), and Group 5 PH to further evaluate the model's robustness across the full PH etiologies. At the same time, patients with other RV diseases, such as RV myocardial infarction, should be included in future cohorts to evaluate the specificity of the observed MCG alterations for PH.

## 6 Conclusion

MCG is a non‐contact, intuitive, and operator‐experience‐independent detection method. This exploratory study extends its application in the field of cardiovascular diseases by enabling the effective differentiation of patients with PH from healthy subjects. In this limited data set, compared to ECG, MCG exhibited superior performance in the detection of PH, particularly concerning the high sensitivity. Although MCG demonstrated promising performance in this small‐sample study, its robustness and real‐world applicability still require further validation through multicenter studies.

## Author Contributions

**Yuankun Qi:** conceptualization, methodology, formal analysis, writing – original draft, writing – review and editing. **Jiaqi Liang:** validation, writing – original draft, writing – review. **Yu Zhang:** formal analysis, software, data curation, writing – original draft. **Jianzhi Yang:** data collection, data curation, writing – review and editing. **Fuzhi Cao:** software, visualization, writing – review and editing. **Xu Zhang:** formal analysis, writing – review and editing. **Haijun Li:** formal analysis, writing – review and editing. **Xiaopei Cui:** conceptualization, methodology, writing – review and editing. **Hongyu Zhang:** conceptualization, project administration, supervision, writing – review and editing. **Min Xiang:** conceptualization, project administration, resources, supervision, writing – review and editing.

## Conflicts of Interest

The authors declare no conflicts of interest related to the MCG device developer. The MCG device manufacturer provided standard operating procedure training and technical assistance, including equipment maintenance.

## Supporting information

Supporting Information S1.

## Data Availability

The data that support the findings of this study are not publicly available due to privacy and ethical restrictions. De‐identified data are available from the corresponding author (Min Xiang, E‐mail: xiang_min@buaa.edu.cn) upon reasonable request.
