# Supplementary material for: Exploring the Application Value of Magnetocardiography in Detecting Pulmonary Hypertension: A Noninvasive and Visual Approach
Source: Clin Cardiol. 2026 Mar 23;49(3):e70277. doi: 10.1002/clc.70277 (PMC13093441; doi:10.1002/clc.70277)
Supplement: Supplementary file 1 — Supporting Information S1. [file CLC-49-e70277-s001.docx]

**Supporting Information**

**Supporting Information Material Methods**

The magnetocardiography (MCG) device was manufactured by Hangzhou National Institute of Extremely-weak Magnetic Field Infrastructure, as illustrated in Figure S1. This device is composed of 32 optically pumped magnetometer (OPM) sensors based on spin exchange relaxation free effect, covering an area of 300 mm × 300 mm, with a sensor spacing of 55 mm. The sensitivity of these sensors is below 50 fT/Hz^1/2^ and the frequency of data acquisition is 1000 Hz. The residual magnetic field inside the semi-open magnetic shielded device does not exceed 50 nT. The raw MCG data were processed through a median filter, a notch filter and a low-pass filter to remove baseline noise, power-line frequency noise, and high-frequency noise, respectively. Subsequently, common-mode noise was eliminated by superposition of the heartbeats. Additionally, independent component analysis (ICA) and empirical mode decomposition (EMD) were employed as necessary.

The workflow of the MCG examination procedure comprises: (1) removaling of metallic and electronic objects; (2) screening for upper-body metallic implants (e.g., orthopedic hardware), non-removable dental prostheses, pacemakers, and claustrophobia; (3) supine positioning with laser-guided alignment; (4) a 90-second data acquisition; and (5) automated report generation. The preparatory steps (1-2) typically require 30-60 s, while positioning and scanning (3-4) take approximately 120 s (including the 90-s recording). With automated reporting completed in ~30 s, the entire clinical workflow is accomplished within 3-4 minutes.

Prior to undergoing at least 90 seconds of MCG measurement, all subjects were instructed to remove any metal jewelry. Furthermore, prior to entering the semi-open magnetic shielded device, laser positioning (mid-sternal, Figure S1) was performed to ensure the uniformity of senor array’s alignment. The “butterfly” diagram, magnetic field (MF) map , and pseudo-current density (PCD) map were generated.

For the purposes of analysis, partial current angles were converted from the range of -180° to 0° to the range of 180° to 360°, as the current angles are conventionally defined from 0° to +180° and -180° to 0°.

Abnormal ECG findings for PH were defined as right axis deviation (RAD), right bundle branch block (RBBB), right ventricular hypertrophy (RVH), RV strain, and P pulmonale, with each parameters binary-coded. The Spiegelhalter-Knill-Jones (SKJ) approach was used to develop an ECG model for PH. Positive likelihood ratios (LR+) and negative likelihood ratios (LR-) were calculated for each ECG finding. A continuity correction of 0.5 was used when zero cells were present. Based on the likelihood ratios, predictor weights were converted into integer points using a scaling factor of 2. Positive and negative point values were assigned to each ECG abnormality. The assigned negative/positive points were -2/4 for RAD, 0/3 for RVH, -1/4 for RBBB, -1/2 for RV strain, and 0/1 for P pulmonale. Finally, the total score of each subjects was entered into a logistic regression model together with age and sex to develop the final model for PH.

**Supplementary Figure**


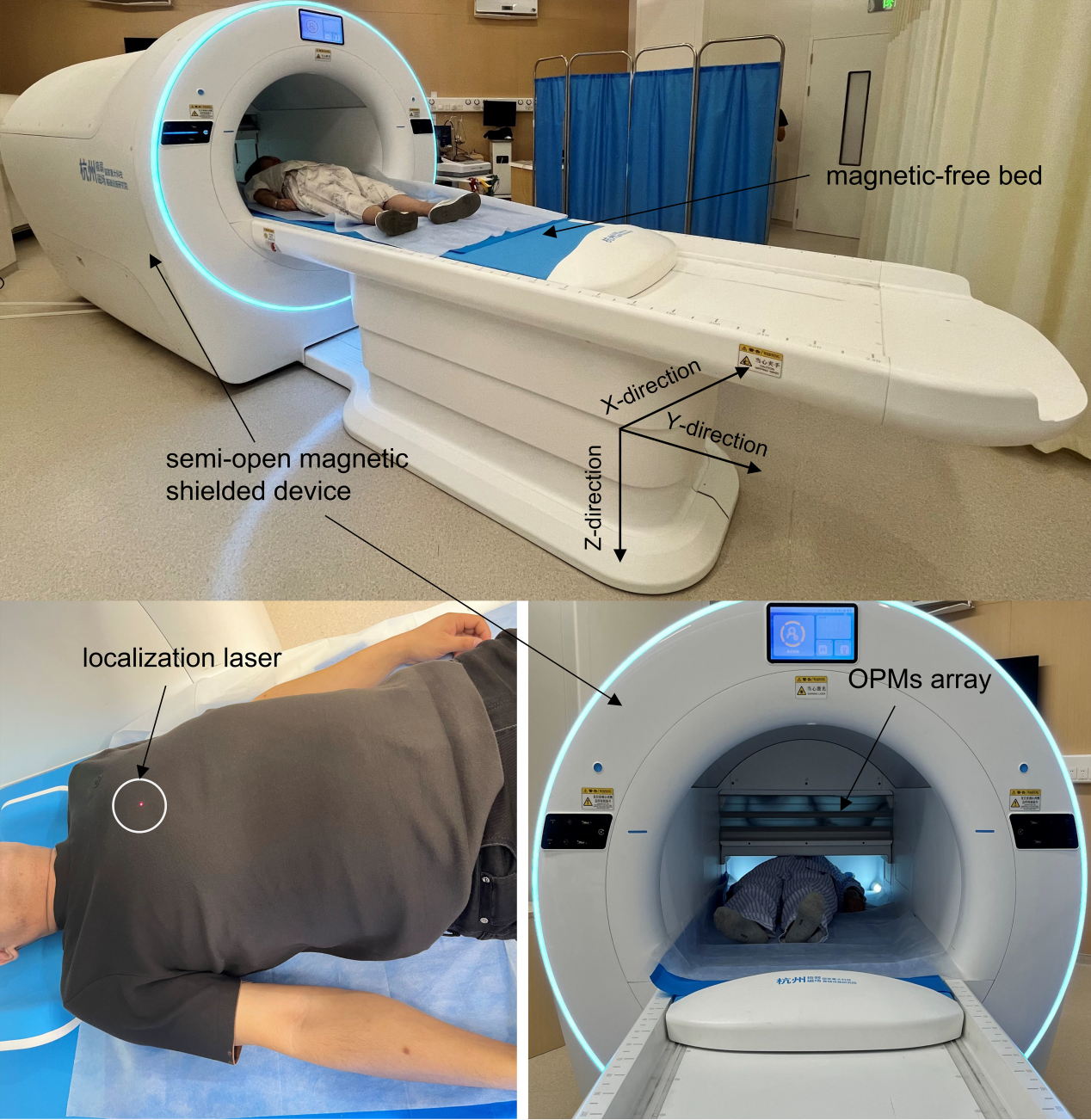


**Figure S1.** Photographs of OPM-based MCG system.

OPM, optically pumped magnetometer


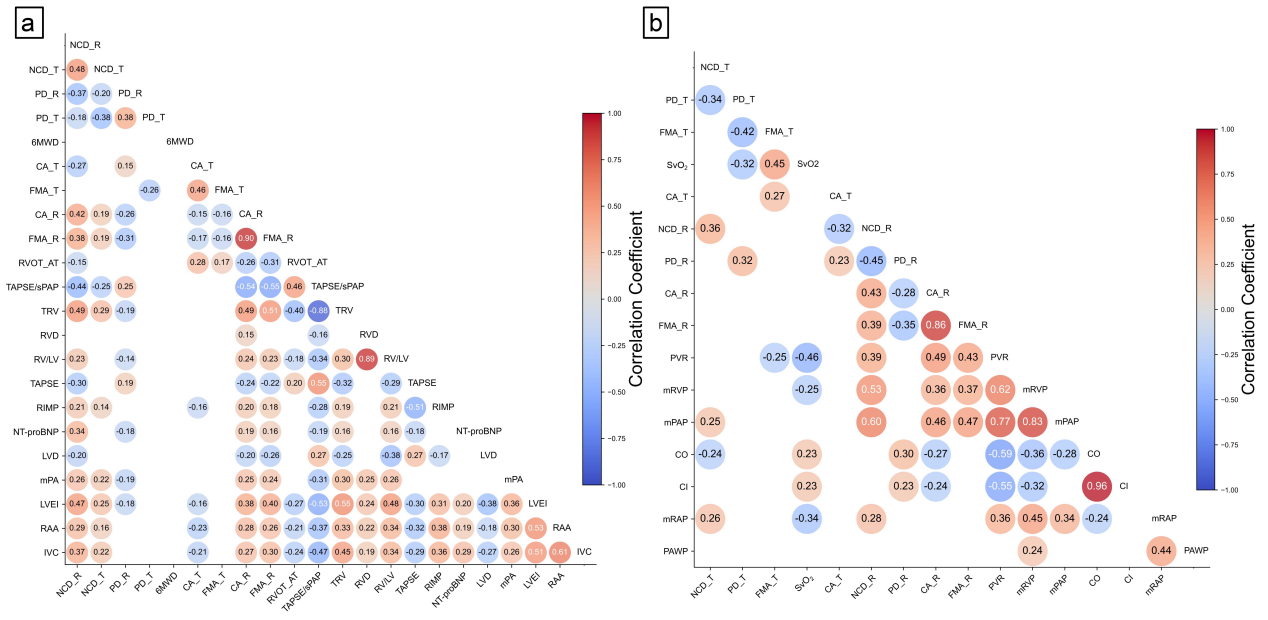


**Figure S2.** The correlationship between MCG and other examinations.

(a) correlation analyses of MCG parameters, TTE parameters, 6MWD and NT-proBNP; (b) correlation analysis of MCG parameters and RHC parameters. CA, current angle; CI, cardiac index; CO, cardiac output; FMA, field map angle; IVC_E_, inferior vena cava diameter of end-expiratory; LVD, left ventricular diameters; LVEI, left ventricular eccentricity index; mPA, main pulmonary arterial diameters; mPAP, mean pulmonary arterial pressure; mRAP, mean right atrial pressure; mRVP, mean right ventricular pressure; NCD, normalized current density; PAWP, pulmonary arterial wedge pressure; PD, poles distance; PVR, pulmonary vascular resistance; RAA, right atrial area; RIMP, right ventricular index of myocardial performance; RVD, right ventricular diameters; RVOT-AT, right ventricular outflow tract-acceleration time; sPAP, systolic pulmonary artery pressure; SvO_2_, mixed venous oxygen saturation; TAPSE, tricuspid annular plane systolic excursion; TRV, tricuspid regurgitation velocity; _R, the moment of R peak; _T, the moment of T peak

**Supplementary Tables**

**Table S1.** MCG and demographic characteristics of training and validation cohort.

| Characteristics | Training cohort  (PH = 103, HC = 147, n = 250) | Testing cohort  (PH = 72, HC = 101, n = 173) | *p* value |
| --- | --- | --- | --- |
| Age, years | 42.08 ± 10.74 | 45.59 ± 12.21 | 0.002 |
| Female, n (%) | 161 (64.4%) | 114 (65.9%) | 0.751 |
| QRSd, ms | 91.94 ± 14.12 | 90.09 ± 14.22 | 0.189 |
| NCD_R | 0.13 (0.08, 0.24) | 0.13 (0.08, 0.25) | 0.474 |
| CA_R, ° | 45.00 (29.00, 105.00) | 44.00 (29.00, 89.50) | 0.690 |
| NCD_T | 0.13 (0.07, 0.25) | 0.14 (0.07, 0.28) | 0.539 |
| CA_T, ° | 25.00 (13.00, 38.00) | 24.13 (11.00, 37.00) | 0.371 |
| PD_R, pixels | 129.40 ± 20.86 | 127.70 ± 20.63 | 0.407 |
| FMA_R, ° | -48.00 (-60.25, 36.00) | -51.00 (-59.00, 30.00) | 0.917 |
| PD_T, pixels | 129.66 ± 21.90 | 127.79 ± 21.96 | 0.389 |
| FMA_T, ° | -65.56 ± 20.34 | -65.46 ± 25.30 | 0.963 |

CA, current angle; FMA, field map angle; NCD, normalized current density; PD, poles distance; _R, the moment of R peak; _T, the moment of T peak.

**Table S2.** The treatment status of PH subgroup B (PH patients in the training cohort).

| characteristics | treatment status |
| --- | --- |
| Time since initial diagnosis, months | 34.00 (15.00, 64.00) |
| PH-specific medications, n (%) |  |
| PDE5i | 9 (8.7%) |
| ERA | 7 (6.8%) |
| sGCs | 1 (1.0%) |
| ERA+PDE5i | 32 (31.1%) |
| ERA+sGCs | 4 (3.9%) |
| ERA+PRA | 3 (2.9%) |
| ERA+PCA | 1 (1.0%) |
| PDE5i+PCA | 1 (1.0%) |
| ERA+PDE5i+PRA | 28 (27.2%) |
| ERA+PDE5i+PCA | 12 (11.7%) |
| ERA+sGCs+PRA | 5 (4.9%) |

ERA, Endothelin Receptor Antagonists; PCA, Prostacyclin Analogues; PDE5i, Phosphodiesterase-5 inhibitor; PRA, Prostacyclin Receptor Agonist; sGCs, Soluble Guanylate Cyclase stimulator

**Table S3.** MCG parameters of different groups

| Parameters | YC | HC | PH | Overall  *p* value ^a^ | *p* (YC vs. HC) ^b^ | *p* (YC vs. PH) ^b^ | *p* (HCs vs. PH) ^b^ |
| --- | --- | --- | --- | --- | --- | --- | --- |
| QRSd, ms | 94.55 ± 15.33 | 91.25 ± 14.30 | 91.09 ± 14.04 | 0.144 | / | / | / |
| NCD_R | 0.12 (0.08, 0.17) | 0.10 (0.06, 0.14) | 0.24 (0.15, 0.37) | < 0.001 | 0.081 | < 0.001 | < 0.001 |
| CA_R, ° | 35.60 ± 14.54 | 32.00 (23.25, 43.75) | 128.00 (69.00, 167.00) | < 0.001 | 1.000 | < 0.001 | < 0.001 |
| NCD_T | 0.16 (0.11, 0.21) | 0.08 (0.05, 0.13) | 0.27 (0.18, 0.36) | < 0.001 | < 0.001 | < 0.001 | < 0.001 |
| CA_T, ° | 33.00 ± 14.57 | 27.50 (18.00, 38.00) | 18.00 (2.00, 37.00) | < 0.001 | 0.244 | < 0.001 | < 0.001 |
| PD_R, pixels | 130.16 ± 18.14 | 140.00 (129.00, 149.00) | 114.00 (108.00, 121.00) | < 0.001 | < 0.001 | < 0.001 | < 0.001 |
| FMA_R, ° | -49.00 (-60.50, -43.50) | -57.00 (-65.00, -51.25) | 47.00 (-2.00, 85.00) | < 0.001 | 0.011 | < 0.001 | < 0.001 |
| PD_T, pixels | 130.00 (118.50, 141.00) | 140.03 ± 18.33 | 113.00 (108.00, 119.00) | < 0.001 | < 0.001 | < 0.001 | < 0.001 |
| FMA_T, ° | -60.85 ± 14.31 | -62.00 (-71.00, -51.00) | -71.98 ± 25.83 | < 0.001 | 1.000 | < 0.001 | < 0.001 |

CA, current angle; FMA, field map angle; QRSd, the duration of the QRS complex; NCD, normalized current density; PD, poles distance; _R, the moment of R peak; _T, the moment of T peak.

^a^, overall *p*-values were calculated using one-way ANOVA (or Kruskal-Wallis H test) across the three groups.

^b^, pairwise comparison *P*-values were strictly adjusted using the Bonferroni correction for multiple testing.

**Table S4.** Predictive value of MCG parameters in testing cohort based on the cut-off values from training cohort

| Parameters | Cut-off  values | Sen  (95% CI) | Spe  (95% CI) | PPV  (95% CI) | NPV  (95% CI) | Acc  (95% CI) | *P* value ^a^ |
| --- | --- | --- | --- | --- | --- | --- | --- |
| NCD_R | > 0.15 | 74.4%  (63.2-83.6%) | 82.1%  (72.9-89.2%) | 77.3%  (66.2-86.2%) | 79.6%  (70.3-87.1%) | 78.6%  (71.7-84.5%) | < 0.001 |
| CA_R, ° | > 60 | 74.4%  (63.2-83.6%) | 96.8%  (91.0-99.3%) | 95.1%  (86.3-99.0%) | 82.1%  (73.8-88.7%) | 86.7%  (80.7-91.4%) | < 0.001 |
| NCD_T | > 0.16 | 75.6%  (64.6-84.7%) | 90.5%  (82.8-95.6%) | 86.8%  (76.4-93.8%) | 81.9%  (73.2-88.7%) | 83.8%  (77.5-89.0%) | < 0.001 |
| CA_T, ° | < 21 | 51.3%  (39.7-62.8%) | 65.3%  (54.8-74.7%) | 54.8%  (42.7-66.5%) | 62.0%  (51.7-71.5%) | 59.0%  (51.2-66.4%) | 0.333 |
| PD_R, pixels | < 127 | 75.6%  (64.6-84.7%) | 80.0%  (70.5-87.5%) | 75.6%  (64.6-84.7%) | 80.0%  (70.5-87.5%) | 78.0%  (71.1-84.0%) | < 0.001 |
| FMA_R, ° | > -30 | 74.4%  (63.2-83.6%) | 95.8%  (89.6-98.8%) | 93.5%  (84.3-98.2%) | 82.0%  (73.6-88.6%) | 86.1%  (80.1-90.9%) | < 0.001 |
| PD_T, pixels | < 121 | 79.5%  (68.8-87.8%) | 88.4%  (80.2-94.1%) | 84.9%  (74.6-92.2%) | 84.0%  (75.3-90.6%) | 84.4%  (78.1-89.5%) | < 0.001 |
| FMA_T, ° | < - 71 | 50.0%  (38.5-61.5%) | 73.7%  (63.6-82.2%) | 60.9%  (47.9-72.9%) | 64.0%  (54.5-73.2%) | 63.0%  (55.3-70.2%) | 0.018 |

Acc, accuracy; CA, current angle; CI, confidence interval; FMA, field map angle; NCD, normalized current density; NPV, negative predictive value; PD, poles distance; PPV, positive predictive value; Sen, sensitivity; Spe, specificity; _R, the moment of R peak; _T, the moment of T peak.

The 95% CIs were calculated using the exact binomial (Clopper-Pearson) method.

^a^, *P*-values were Bonferroni-adjusted to control the family-wise error rate across the evaluated parameters.

**Table S5** The results of collinearity diagnostics.

| Parameters | Tolerance | VIF |
| --- | --- | --- |
| Initial model |  |  |
| Sex | 0.767 | 1.304 |
| Age, years | 0.836 | 1.196 |
| CA_R, ° | 0.074 | 13.551 |
| NCD_T | 0.315 | 3.171 |
| PD_R, pixels | 0.437 | 2.290 |
| FMA_R, ° | 0.073 | 13.735 |
| PD_T, pixels | 0.407 | 2.457 |
| FMA_T, ° | 0.334 | 2.992 |
| Final model |  |  |
| Sex | 0.918 | 1.090 |
| Age, years | 0.950 | 1.053 |
| CA_R, ° | 0.593 | 1.685 |
| NCD_T | 0.584 | 1.711 |
| PD_R, pixels | 0.584 | 1.712 |

VIF, vriance inflation factors

**Table S6.** The comparison of MCG models based on CA_R and FMA_R.

| Models ^a^ | -2 LL | AUC (95% CI) |
| --- | --- | --- |
| Based on the CA_R | 72.979 | 0.985 (0.971-0.998) |
| Based on the FMA_R | 78.648 | 0.984 (0.972-0.997) |

-2 LL, -2 Log Likelihood.

^a^, based on fixed 9 parameters, CA_R and FMA_R were added to the model separately for evaluation. The logistic regression modeling using “enter” method.

**Table S7**. The complete model with all original parameters.

| Parameters | B | OR (95% CI) | *p*-value |
| --- | --- | --- | --- |
| Sex | -1.813 | 0.163 (0.029-0.902) | 0.038 |
| Age | 0.005 | 1.005 (0.939-1.076) | 0.878 |
| NCD_T | 0.187 | 1.205 (1.073-1.353) | 0.002 |
| PD_R, pixels | -0.080 | 0.923 (0.881-0.967) | < 0.001 |
| CA_R, ° | 0.903 | 2.446 (1.639-3.712) | < 0.001 |
| QRSd, ms | -0.009 | 0.991 (0.940-1.046) | 0.750 |
| NCD_R | -0.010 | 0.990 (0.966-1.015) | 0.432 |
| CA_T, ° | -0.067 | 0.935 (0.870-1.006) | 0.072 |
| PD_T, pixels | 0.024 | 1.024 (0.973-1.078) | 0.356 |
| FMA_T, ° | 0.054 | 1.056 (0.989-1.127) | 0.103 |
| Constant | 5.596 |  | 0.329 |

**Table S8.** The distribution of ECG findings across different PH etiologies.

| PH etiologies(n = 175) | RAD | RVH | RBBB | RV strain | P pulmonale |
| --- | --- | --- | --- | --- | --- |
| I/HPAH | 41 (23.4%) | 13 (7.4%) | 18 (10.3%) | 24 (13.7%) | 3 (1.7%) |
| CHD-PAH | 42 (24.0%) | 12 (6.9%) | 29 (16.6%) | 38 (21.7%) | 4 (2.3%) |
| CTD-PAH | 14 (8.0%) | 5 (2.9%) | 3 (1.7%) | 14 (8.0%) | 1 (0.6%) |
| PoPH | 1 (0.6%) | 0 | 0 | 1 (0.6%) | 0 |
| PVOD/PCH-PAH | 2 (1.1%) | 1 (0.6%) | 1 (0.6%) | 2 (1.1%) | 0 |
| CTEPH | 11 (6.3%) | 2 (1.1%) | 4 (2.3%) | 16 (9.1%) | 1 (0.6%) |
| Total | 111 (63.4%) | 33 (18.9%) | 55 (31.5%) | 95 (54.3%) | 9 (5.1%) |

CHD-PAH, congenital heart disease associated pulmonary arterial hypertension; CTD-PAH, connective tissue disease associated pulmonary arterial hypertension; CTEPH, chronic thromboembolic pulmonary hypertension; I/HPAH, idiopathic or heritable pulmonary arterial hypertension; PoPH, porto-pulmonary hypertension; PVOD/PCH-PAH, pulmonary arterial hypertension associated with pulmonary veno-occlusive disease or capillary haemangiomatosis; RAD, right axis deviation; RBBB, right bundle branch block; RVH, right ventricular hypertrophy; RV strain, right ventricular strain.

**Table S9.** The results of McNemar’s exact test for paried sensitivity and specificity analyses.

| The results of MCG | ECG (+) | ECG (-) |
| --- | --- | --- |
| PH patients comparison for sensitivity |  |  |
| MCG (+) | 127 | 33 |
| MCG (-) | 1 | 14 |
| Non-PH patients comparison for specificity |  |  |
| MCG (+) | 0 | 2 |
| MCG (-) | 1 | 10 |

For sensitivity, the McNemar exact *p* < 0.001; and for specificity, the McNemar exact *p =* 1.000.
